# A Case Report of a Mysterious Mycetoma

**DOI:** 10.7759/cureus.64608

**Published:** 2024-07-15

**Authors:** Tammy Luan, Madison R Guido, Majid Chammas, Javier Valdes, Nicholas S Cortolillo

**Affiliations:** 1 Department of Surgery, University of Miami JFK Medical Center, Atlantis, USA; 2 Department of Surgery, University of Miami Miller School of Medicine, Miami, USA

**Keywords:** mycetoma medical management, mycetoma surgical management, special stains for mycetoma, eumycetoma, actinomycetoma, management of mycetoma, neglected tropical disease, cutaneous nocardia infection, mycetoma

## Abstract

Mycetoma, a chronic subcutaneous infection caused by bacterial or fungal species from soil and water, presents a diagnostic challenge due to its rarity and diverse clinical manifestations. Predominantly affecting male workers in endemic regions, mycetoma typically manifests as painless swelling evolving into purulent lesions with draining sinuses in the extremities. Although historically uncommon in regions like North America, rising immigration and international travel have led to an increased prevalence, necessitating heightened clinical suspicion. Early diagnosis is crucial to prevent severe complications such as limb loss and septicemia. This case report details the diagnosis and management of chronic actinomycetoma due to *Nocardia spp.* in a Guatemalan immigrant landscaper and emphasizes the importance of comprehensive understanding and timely intervention in mycetoma cases.

## Introduction

Mycetoma is a chronic subcutaneous infection caused by bacterial or fungal species found in soil and water. Mycetoma infections are characterized by the formation of inflammatory granulomas in affected tissues, which consist of immune cells like macrophages surrounded by lymphocytes and fibroblasts due to persistent infection or foreign substances [1]. These bacterial and fungal pathogens typically enter the subcutaneous tissue via transcutaneous inoculation and then propagate [2]. Infection is commonly seen following trauma to the skin barrier with splinters, thorns, or other plant debris [3]. Mycetoma predominantly affects men and is often associated with specific occupations, commonly observed among farmers, field laborers, landscapers, herders, and other individuals whose work involves frequent contact with soil [4-5].

Infections causing mycetoma can persist and spread over the course of months to years. Clinically, mycetoma initially presents as painless swelling, evolving into purulent lesions with fistulas that can extend to deep anatomical structures, including muscles, tendons, joints, fascia, and bones. These inflammatory fistulas often exhibit exudative drainage with granules of varying sizes, representing microcolonies of the causative agent [3-6]. Mycetoma most frequently manifests in the extremities, particularly the foot, but may also develop in other regions of the body, such as the shoulder and back [1,3]. Typically, there are no associated systemic symptoms or adenopathy [4]. 

There have been over 70 organisms found to cause mycetoma [5]. The majority of mycetoma infections are caused by fungi or actinomycetes, resulting in eumycetoma and actinomycetoma, respectively [1]. The distribution of cases is approximately 60% actinomycetoma and 40% eumycetoma [6]. The primary bacterial species responsible for actinomycetoma are *Nocardia brasiliensis*, *Streptomyces somaliensis*, and *Actinomadura madurae* [3]. Conversely, the prevalent eumycetoma species include *Madurella mycetomatis*, *Madurella grisea*, and *Scedosporium apiospermum* [4].

Historically, mycetoma has been a rare occurrence in the United States (USA) [1]. Categorized as a neglected tropical disease by the World Health Organization (WHO) due to inadequate global surveillance and the absence of control programs, mycetoma is often misunderstood, misdiagnosed, and mismanaged [7,8]. Although mycetoma is most frequent in remote rural areas of tropical and subtropical regions like Africa, South America, and South Asia, its clinical prevalence in the USA has been on the rise due to increased immigration and international travel in recent years [1,5,9]. Therefore, it is imperative that clinicians maintain a high index of suspicion for mycetoma in patients from endemic regions that are presenting with painless or painful masses in the extremities. Comprehensive knowledge of the epidemiology, risk factors, and clinical presentation of this disease is crucial, as it can facilitate earlier diagnosis and prompt treatment, preventing severe complications such as limb loss, septicemia, and mortality. We present a distinctive case illustrating the diagnosis and management of chronic actinomycetoma in an immigrant from Guatemala employed as a landscaper.

## Case presentation

A 56-year-old otherwise healthy Guatemalan male landscaper presented to the emergency room with complaints of worsening swelling and pain in his right lower extremity over the past two months, accompanied by multiple raised and indurated lesions at various stages of healing along the right posteriomedial calf. In 2018, the patient experienced a similar episode involving the right lower extremity, during which surgical debridement and primary closure by podiatry were performed. This was followed by a two-week course of trimethoprim-sulfamethoxazole (TMP-SMX) and cefazolin, resulting in complete resolution of symptoms related to coexisting *Nocardia spp.*, methicillin-susceptible* Staphylococcus aureus* (MSSA), and *Streptococcus agalactiae *group B infections as confirmed by blood and tissue cultures at that time.

**Figure 1:**
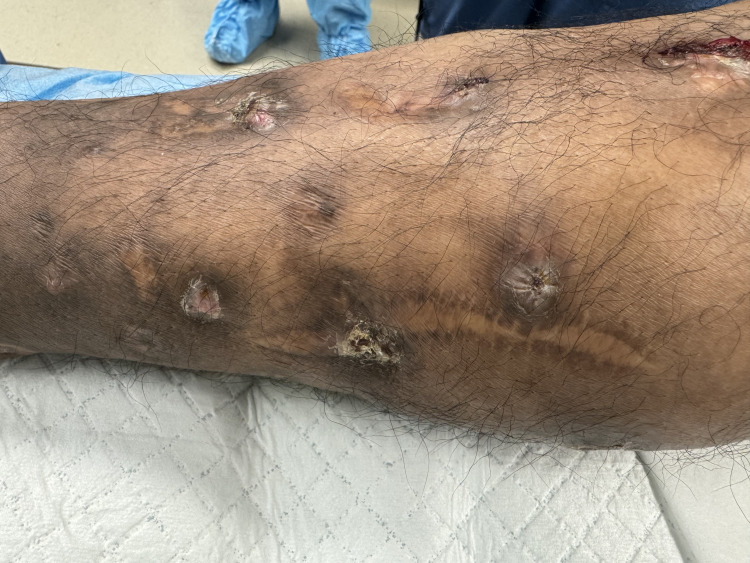
Image of the right medial calf showing several raised and indurated lesions in various stages of healing. The posteriomedial scar is from a surgical debridement completed in 2018 due to a previous mycetoma

**Figure 2:**
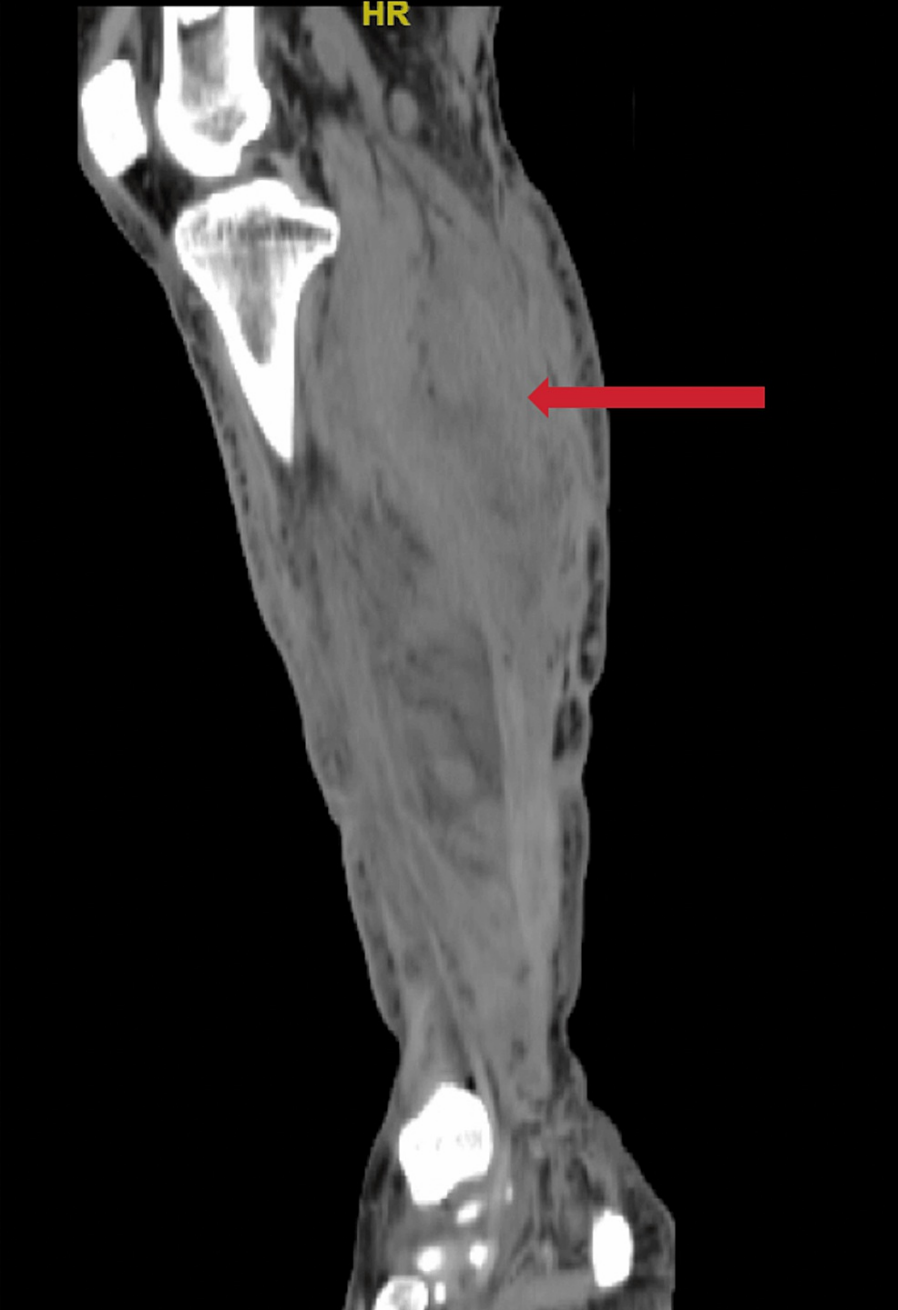
CT scan of the right distal lower extremity with diffuse subcutaneous fat stranding and a heterogeneous collection in the posteriomedial calf as indicated by the red arrow CT: Computed tomography

The physical exam during his current presentation revealed marked edema of the distal right lower extremity, multiple granulomatous foci, and two well-healed scars along the medial and lateral aspects of the calf, attributable to his previous surgical debridement from 2018 (Figure 1). He had full strength and sensation in the affected extremity without any discernible deficits. There were no signs indicative of a systemic infection, and the patient’s vital signs remained stable. A computed tomography (CT) scan was completed, showing superficial compartment edema of the posterior distal lower extremity, along with an irregular heterogenous collection in the posteriomedial calf (Figure 2). Given the uncertainty regarding deep compartment involvement and the high suspicion of a developing abscess due to a recurrent *Nocardia spp.* infection, the patient was subsequently taken to the operating room. 

**Figure 3:**
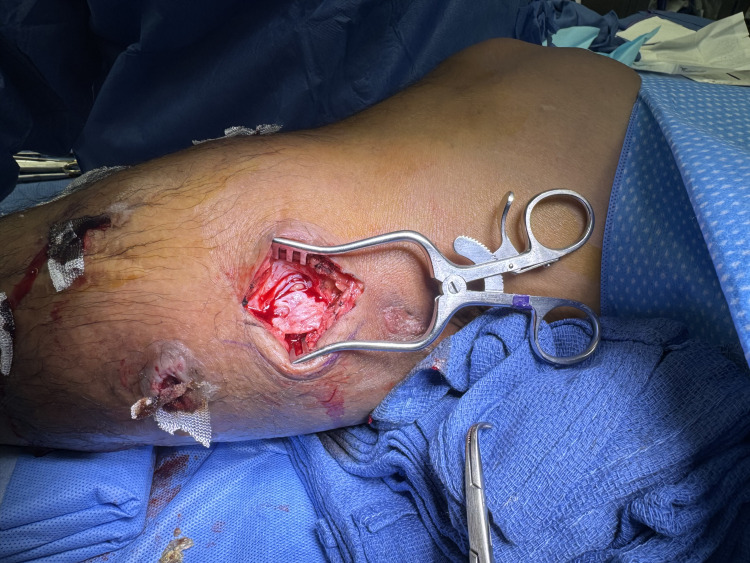
Image showing a cutaneous lesion on the posteriomedial calf prior to surgical excision

**Figure 4:**
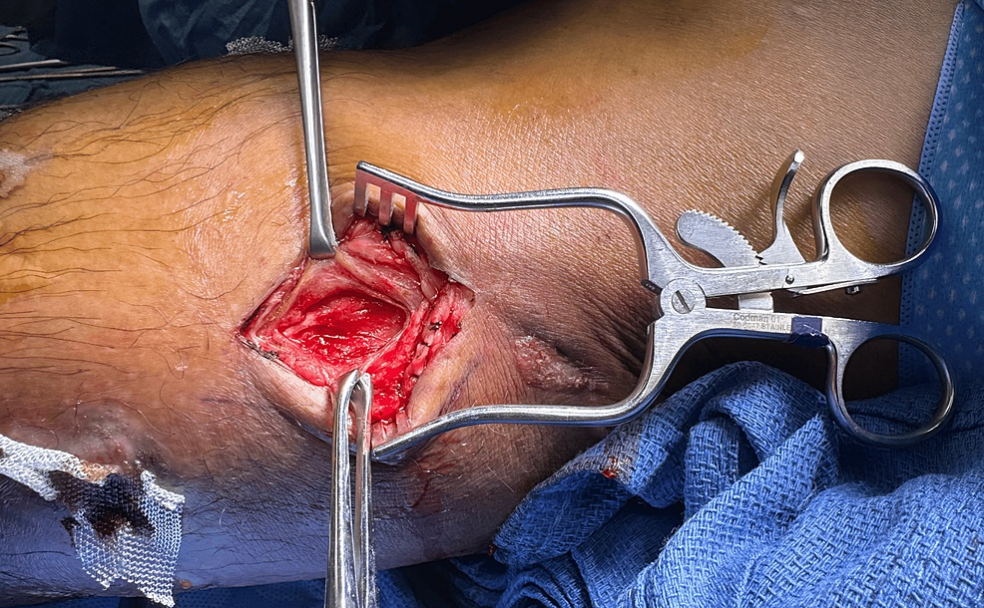
Image showing a cutaneous lesion on the posteriomedial calf following surgical excision, with visualization of healthy deep tissue

In the operating room, an incisional biopsy of the cutaneous lesions was conducted to confirm the identity of the infective species and to surgically explore for potential involvement of deeper leg structures (Figure 3). During surgery, sharp excisional debridement was performed on 12 lesions, with deeper lesions requiring excision into the subcutaneous fat until healthy tissue was exposed (Figure 4). Excised specimens were sent for culture and permanent pathology.

An ultrasound (US) was then used to visualize the compartments of the lower extremity, revealing the presence of a subfascial abscess within the medial compartment of the gastrocnemius. A surgical incision was made through the skin, subcutaneous fat, and muscle, revealing muscle fascia surrounding the abscess that appeared thickened, hardened, and white in color. A generous, sharp excision of the sclerotic muscle and intramuscular abscess was performed. Approximately 2 ml of purulent fluid was drained from the abscess and sent for culture. The muscular specimen was sent for permanent pathology. 

The final surgical pathology from the biopsied lesions and muscle indicated small clusters of thin filiform bacteria, which stained positively for Gram and Ziehl-Neelsen, most consistent with an actinomycetoma. No molecular testing or sequencing was completed. The patient was placed on double-strength TMP-SMX 800-160 mg for *Nocardia spp.* coverage for three months with no complications and complete resolution of symptoms. 

## Discussion

Mycetoma is a relatively rare and obscure disease that can lead to devastating complications, including limb loss, sepsis, and death [2,5]. It is predominantly observed in tropical regions; however, there has been a rising number of reported cases in the USA in recent years [1,7,9]. Given the potential for severe complications and the increasing prevalence in the USA, it is imperative for physicians, including surgeons and dermatologists, to maintain a high level of suspicion for mycetoma in patients from endemic regions who are presenting with clinical symptoms such as swelling or lesions in the extremities. 

Mycetoma predominantly occurs in male rural workers, commonly affecting anatomical regions like the feet and hands [3,5]. Clinically, it initially presents as painless subcutaneous masses and progresses over time to form multiple inflammatory sinuses with purulent discharge containing grains [1,4]. Given the often painless nature of early symptoms, patients may frequently delay seeking medical attention for mycetoma, allowing the condition to progress considerably [5,8]. This further underscores the crucial importance of early diagnosis and treatment to improve patient outcomes. In our patient's case, no draining sinus tracts were observed, but numerous subcutaneous masses were evident along his right lower extremity. His severe pain was attributed to a mass effect caused by significant inflammation and edema, which was ultimately the factor that prompted him to seek medical attention. 

Imaging modalities such as US or magnetic resonance imaging (MRI) serve as noninvasive techniques for early diagnosis of mycetoma. Both US and MRI depict a distinctive "dot-in-circle" sign characteristic of mycetoma, where the circular lesions correspond to granulomatous aggregates and the small dots represent bacterial or fungal grains [10-11]. In recent studies, MRI has emerged as the preferred imaging modality due to its exceptional sensitivity and ability to delineate soft tissue contrast. On MRI, mycetoma infections appear as high-intensity foci with hypo-intense centers, surrounded by low-intensity peripheral rings, which, respectively, represent the granulomas, fungal or bacterial grains, and fibrotic tissue [11].

Mycetoma can also be diagnosed by examining granules found in the exudate draining from sinus tracts [3]. In cases such as our patient's, where no sinus tracts are present, performing a fine-needle aspiration or incisional biopsy is necessary to obtain a sample containing grains [4,12]. Grains in eumycetoma and actinomycetoma vary in size, color, and texture [3-4]. Actinomycetoma grains, measuring less than 1 µm, consist of thin filaments visible only under a microscope. Common colors for actinomycetoma grains include red, yellow, or white. In contrast, eumycetoma grains are larger, exceeding 2 µm, and possess thicker, coarser filaments that often appear black in color, though variations of white or yellow are also observed [4-5,13]. 

Special stains are also essential in identifying the causative organisms in mycetoma cases. Gram staining distinguishes between actinomycetoma, caused by Gram-positive bacteria, and eumycetoma, caused by fungi [6,12]. Histologically, hematoxylin and eosin (H&E) staining commonly reveals suppurative granulomas surrounding grains in the subcutaneous tissue, often accompanied by multiple giant cells, which is characteristic of mycetoma. Additionally, similar to Gram staining, H&E staining identifies thin filamentous structures encircled by eosinophilic fringe in actinomycetoma and thick, round, occasionally oval, or kidney-like configurations in eumycetoma [4,6]. Ziehl-Neelsen staining serves as a valuable tool for distinguishing specific actinomycotic species, whereas Gomori methenamine silver or periodic acid-Schiff stains are more adept at differentiating species that cause eumycetoma [4,6,12]. Finally, cultures followed by subsequent sensitive molecular tests like 16S rRNA gene sequencing, internal transcribed spacer (ITS) gene sequencing, or matrix-assisted laser desorption ionization-time-of-flight mass spectrometry (MALDI-TOF MS) are definitive for exact bacterial or fungal genus and species identification [4,6].

Mycetoma is treated comprehensively with a combination of surgical excision and extensive pharmacotherapy. Surgical debridement alone leads to a 90% recurrence rate of mycetoma [7]. For actinomycetoma cases like our patient's, first-line antibiotics include amikacin (15 mg/kg/day) for three weeks alongside TMP-SMX (40/8 mg/kg/day) twice daily for five weeks, or surgical excision if the condition is more advanced. In cases of resistance or allergies to this combination, amoxicillin-clavulanate can replace TMP-SMX, netilmicin can substitute for amikacin, or amikacin can be paired with a carbapenem [2,4]. In treating eumycetoma, itraconazole stands as the primary pharmacotherapeutic choice. While certain case studies have shown promising results with voriconazole, posaconazole, and terbinafine, further clinical data is required to substantiate their efficacy [2,4,13]. Finally, it is crucial to remain mindful of potential medication side effects and to conduct thorough screenings, including appropriate lab tests and imaging, prior to initiating treatment.

## Conclusions

Mycetoma, although rare, demands increased awareness within the medical community, particularly in regions like North America where it remains less recognized. Physicians should be vigilant when patients from endemic regions present with lesions or sinus tracts on the extremities, as untreated cases can lead to severe consequences such as disability or death. Familiarity with diagnostic and treatment protocols is crucial for improving outcomes in these cases. Surgical management alone is not enough to treat and prevent the recurrence of mycetoma. Careful selection of long-term pharmacotherapy suitable to treat the causative organism of the mycetoma is also essential.
